# Two Unusual Gastrointestinal Foreign Bodies

**DOI:** 10.5402/2011/187343

**Published:** 2011-06-08

**Authors:** Ahmed H. Al-Salem

**Affiliations:** Department of Pediatric Surgery, Maternity and Children Hospital, Dammam 31911, P.O. Box 61015, Qatif, Saudi Arabia

## Abstract

Swallowed foreign bodies are common in the pediatric age group, but fortunately, the majority of them pass spontaneously without any adverse effects. Tube gastrostomy is an excellent method to provide prolonged enteral feeding. It is, however, associated with complications, namely, intraperitoneal leak and distal migration of the gastrostomy tube causing gastric outlet obstruction. This paper describes two unusual gastrointestinal foreign bodies, one was swallowed, while the other one was a complication of a tube gastrostomy.

## 1. Introduction

Swallowed foreign bodies are a common problem in the pediatric age group. Fortunately, the majority of them traverse the gastrointestinal tract without any adverse effects [1–3]. A large variety of foreign bodies can be swallowed by children including some bizarre foreign bodies [4]. This paper describes two unusual foreign bodies in the gastrointestinal tract, one was swallowed while the other one was a complication of a tube gastrostomy. The hazards of foreign bodies, diagnostic dilemma and management are also highlighted.

## 2. Case Reports

**Case No. 1**

**Figure 1:**
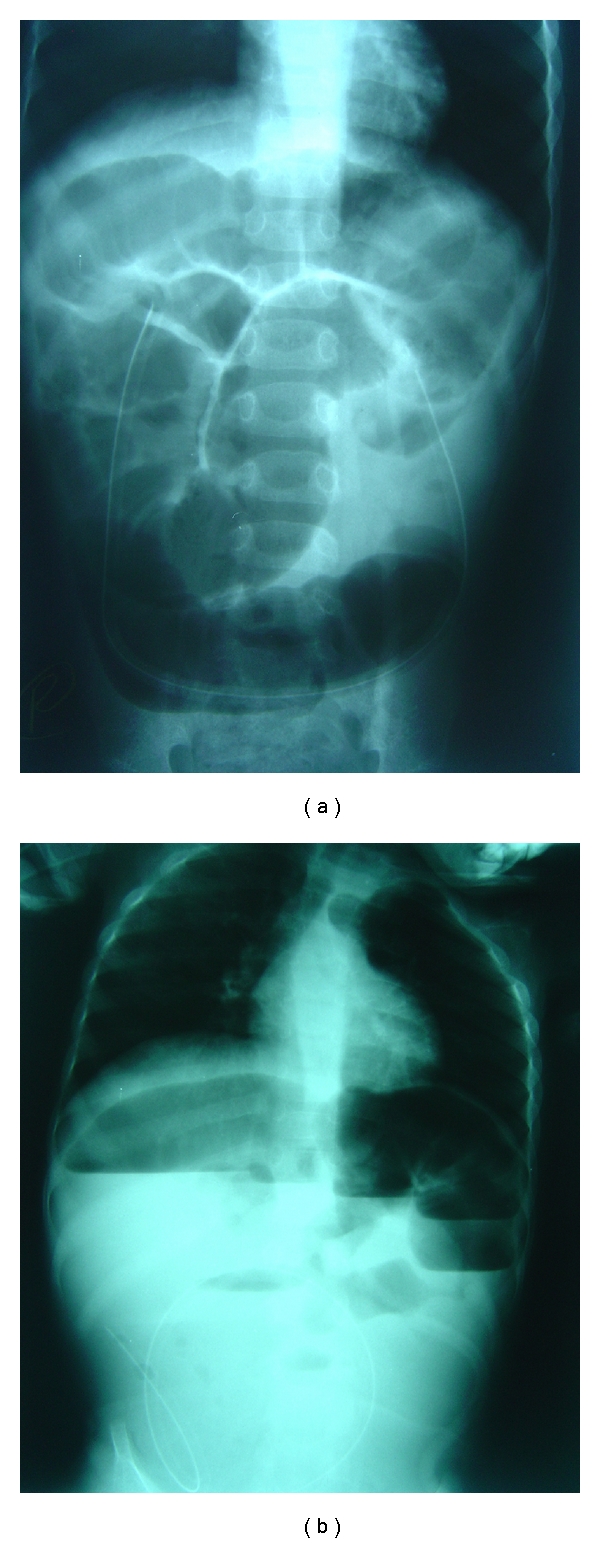
Plain supine and erect abdominal X-rays showing dilated small bowel loops with multiple air-fluid levels. Note the remaining portion of the Foley's catheter.

**Figure 2:**
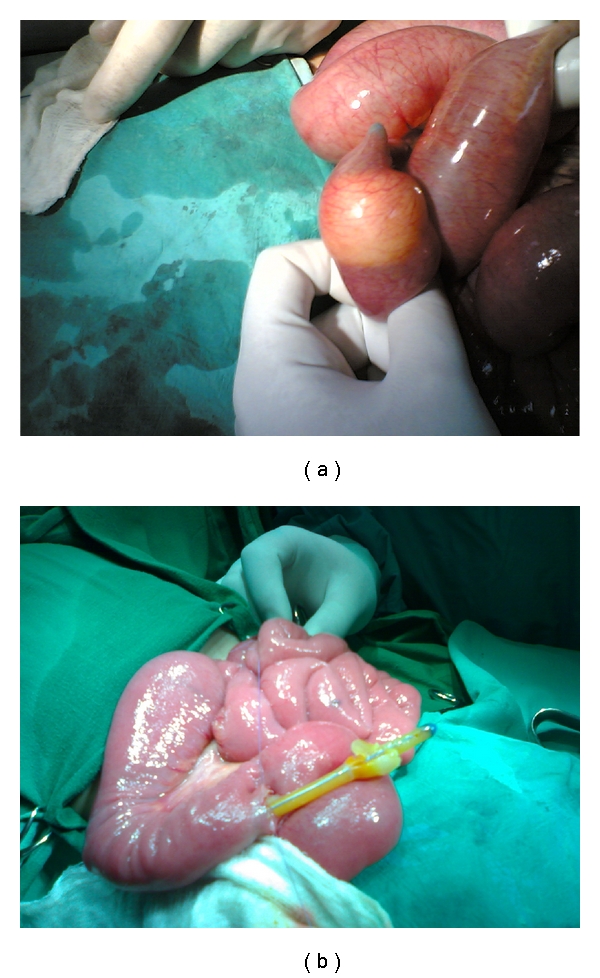
Intraoperative photographs showing the Foley's catheter with its balloon not deflated (a) and after it was deflated (b).

**Figure 3:**
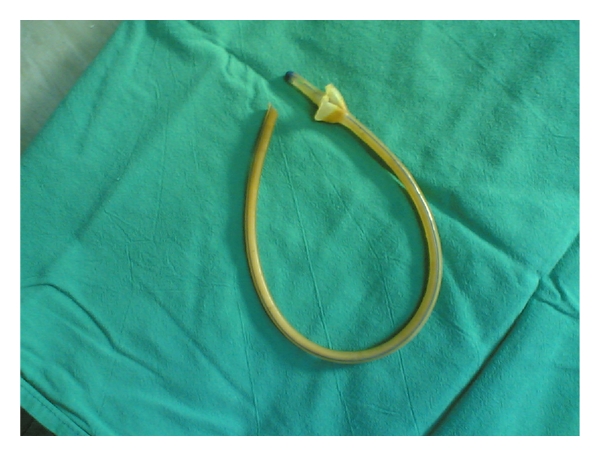
The Foley's catheter after it was removed. Note the punctured balloon.

A 5-year-old male, a known case of cerebral palsy, underwent Nissen's fundoplication and feeding gastrostomy. He presented to our hospital several months later, because the gastrostomy Foley's catheter was accidentally cut. The mother was concerned about the remaining portion of the Foley's catheter which was left behind. He was admitted to the hospital and underwent gastroscopy via the gastrostomy. This, however, revealed no foreign body in the stomach. The patient was asymptomatic, and so, he was discharged home hoping that the remaining portion of the Foley's catheter will pass spontaneously. On the same day in the evening, he was readmitted with bilious vomiting and abdominal distension. Plain abdominal X-ray erect and supine revealed dilated small bowel loops with multiple air-fluid levels with the Foley's catheter lying within the bowel loops (Figures 1(a) and 1(b)). The possibility of a twist of a small bowel loop around the loop with the Foley's catheter was suspected. The patient was resuscitated and underwent exploration laparotomy. This revealed multiple dilated small bowel loops with the Foley's catheter stuck in the ileum causing its obstruction with the balloon that, in spite of the catheter was cut, did not deflate (Figures 2(a) and 2(b)). Via a small enterotomy, the balloon was deflated, and the catheter was removed (Figure 3). Postoperatively, the patient did well and was discharged home in a good general condition.

**Case No. 2**

**Figure 4:**
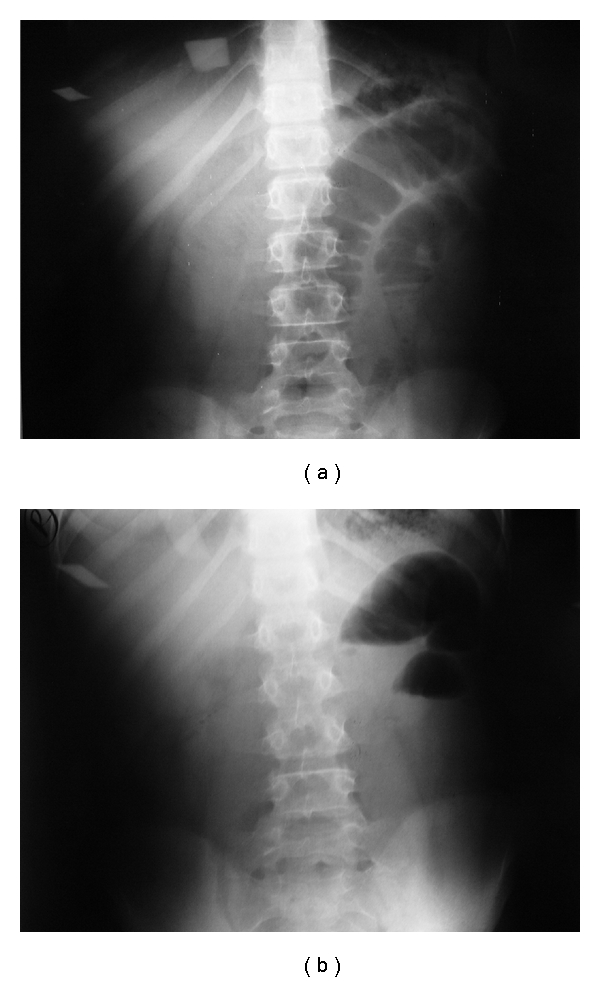
Plain abdominal X-rays showing dilated loops with multiple air fluid levels.

**Figure 5:**
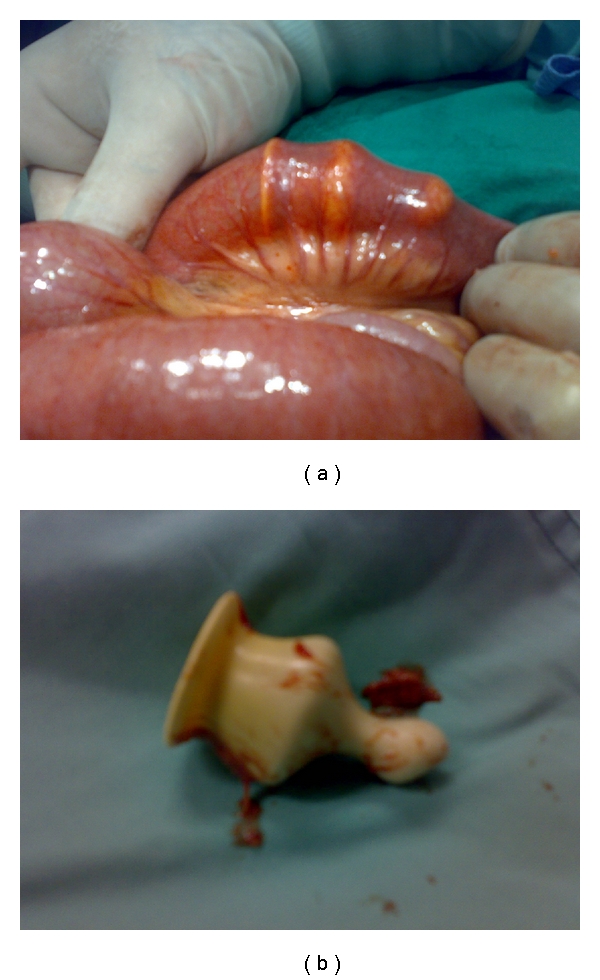
Intraoperative photograph showing the pacifier inside the small intesines (a) and after it passed (b).

**Figure 6:**
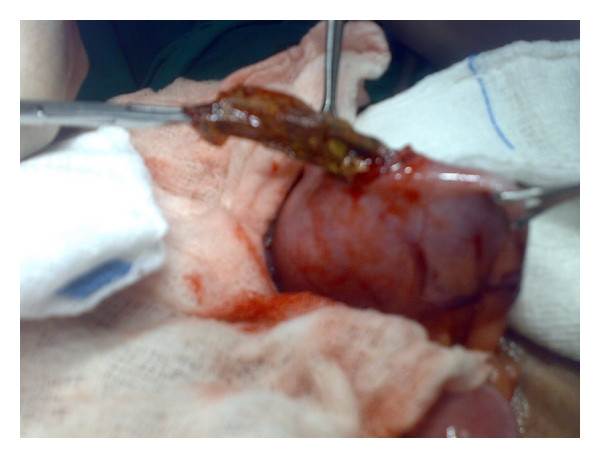
Clinical photograph showing the plastic bag removed from the stomach.

A 10-year-old male child was admitted to the hospital with bile-stained vomiting and colicky abdominal pain of two-day duration. There was no history of previous surgery. Clinically, he was a normal-looking well-developed child with mild abdominal distension, mild diffuse tenderness and, active bowel sounds. Plain abdominal X-rays revealed few dilated small bowel loops with multiple air-fluid levels (Figures 4(a) and 4(b)). The possibility of malrotation was suspected, and he underwent exploration laparotomy. This revealed dilated small bowel loops with obstruction at the lower ileum caused by a foreign body (Figure 5(a) and 5(b)). This was a large pacifier. It was milked through the ileocecal valve into the colon and then extracted via the anus. Prior to closure of the wound, palpation of the stomach revealed a strange large foreign body. This was removed via a small gastrotomy and shown to be a plastic bag (Figure 6). Postoperatively, the patient did well and was discharged home in a good general condition. Prior to discharge, he admitted that because of jealousy from his younger brother, he swallowed the pacifier with its plastic bag.

## 3. Discussion

A large variety of foreign bodies are swallowed by children, but the majority, however, passes through the gastrointestinal tract without any adverse effects [1–4]. The highest incidence of swallowed foreign bodies occurs in children between 6 months and 3 years, and coins are the most commonly ingested foreign bodies [3, 4]. Although 80% to 90% of swallowed foreign bodies will pass spontaneously, there is a definite predilection for swallowed foreign bodies to become stuck at the level of cricopharyngeus and just below it or at the esophagogastric junction [5]. In our second patient and in spite of the large size of the swallowed foreign body, it passed with its plastic bag through the esophagus and into the stomach. In the stomach, the plastic bag opened, and to our surprise, the large foreign body passed through the pylorus to cause acute intestinal obstruction in the jejunum. The size of the jejunum is larger than that of the pylorus and duodenum and for this foreign body to pass through the pylorus; it should have passed through the whole small intestines to become stuck at the level of the ileocecal valve. We managed to milk it all the way through the small intestines and colon. The plastic bag stayed in the stomach and was removed via a small gastrostomy. The swallowed foreign body was not visible on plain abdominal X-rays, and it was a diagnostic dilemma for us. Although rare at this age, the possibility of a swallowed foreign body must always be kept in mind as a possible cause of acute intestinal obstruction.

Tube gastrostomy is one of the common operations performed for prolonged enteral feeding especially in neurologically impaired children. It is, however, associated with complications. Two common complications of tube gastrostomy are intraperitoneal leak leading to peritonitis and distal migration of the gastrostomy tube producing gastric outlet obstruction [6, 7]. Other rare and an unusual complications include perforation of the stomach, esophagus, duodenum, intussusceptions, and stomach prolapse at the gastrostomy site [8–11]. In our patient, the gastrostomy tube was cut, and the distal part migrated passing through the pylorus to cause small bowel obstruction. The Foley's catheter, in spite of being cut, the balloon did not deflate and migrated intact to pass through the pylorus. The catheter's balloon did not deflate possibly because of manufacturing fault or improper storage. One way to obviate this complication is to use a button gastrostomy if available instead of Foley's catheter. This is also more convenient to the patients and parents.
